# Wavelet Packet Feature Assessment for High-Density Myoelectric Pattern Recognition and Channel Selection toward Stroke Rehabilitation

**DOI:** 10.3389/fneur.2016.00197

**Published:** 2016-11-21

**Authors:** Dongqing Wang, Xu Zhang, Xiaoping Gao, Xiang Chen, Ping Zhou

**Affiliations:** ^1^Department of Electronic Science and Technology, University of Science and Technology of China, Hefei, China; ^2^Department of Rehabilitation Medicine, First Affiliated Hospital of Anhui Medical University, Hefei, China; ^3^Department of Physical Medicine and Rehabilitation, University of Texas Health Science Center at Houston, Houston, TX, USA; ^4^TIRR Memorial Hermann Research Center, Houston, TX, USA; ^5^Guangdong Work Injury Rehabilitation Center, Guangzhou, China

**Keywords:** myoelectric control, pattern recognition, wavelet packet transform, channel selection, stroke rehabilitation

## Abstract

This study presents wavelet packet feature assessment of neural control information in paretic upper limb muscles of stroke survivors for myoelectric pattern recognition, taking advantage of high-resolution time–frequency representations of surface electromyogram (EMG) signals. On this basis, a novel channel selection method was developed by combining the Fisher’s class separability index and the sequential feedforward selection analyses, in order to determine a small number of appropriate EMG channels from original high-density EMG electrode array. The advantages of the wavelet packet features and the channel selection analyses were further illustrated by comparing with previous conventional approaches, in terms of classification performance when identifying 20 functional arm/hand movements implemented by 12 stroke survivors. This study offers a practical approach including paretic EMG feature extraction and channel selection that enables active myoelectric control of multiple degrees of freedom with paretic muscles. All these efforts will facilitate upper limb dexterity restoration and improved stroke rehabilitation.

## Introduction

Restoration of upper limb function is an important but challenging task in stroke rehabilitation due to arm/hand dexterity (which is critical for daily activities). A number of upper limb robotic devices have been designed to assist rehabilitation training for promoting upper limb motor recovery (1, 2) among which some recently emerging ones involve different human–machine interfaces enabling active response to user’s intention. Compared with passive training, such an active control approach has proven to be more effective for motor function improvement (3, 4).

Electromyogram (EMG) is one of the most commonly used control signals for artificial limbs, rehabilitation robots, and other assistive devices (5–7). Most development in myoelectric control is primarily based on a simple control strategy that the EMG of a single muscle is mapped to a single degree of freedom (DOF). Considering the complexity of upper limb functional movements performed by multiple muscles, it is unfeasible to control multiple DOFs through such a straightforward mapping (8). Because of this, myoelectric pattern recognition has been developed for controlling of multiple DOFs (8–10). So far, the myoelectric pattern recognition control strategy has been primarily focused on improving dexterity of prosthesis control for amputee users, whereas its application for neurological injury patients has not been fully explored (5). Only very recently, myoelectric pattern recognition was first reported to detect movement intention of affected limb after stroke (8). A more comprehensive assessment of neural control information from paretic muscles of stroke subjects was further performed using high-density surface EMG recording and pattern recognition techniques (4).

While high-density surface EMG pattern recognition has revealed substantial neural control information that can be extracted from neurologically impaired muscles, there are a number of issues to be considered for developing a myoelectric control system. These include assessment of different EMG features, selection of a practical number of appropriate EMG channels (myoelectric control sites), and user-specific design according to individual need and performance. A variety of features describing surface EMG signals in different (time, frequency, time–frequency, etc.) domains have been used for myoelectric pattern recognition analysis, but primarily aimed at prosthetic control (9–12). So far for patients with neurological injuries, the myoelectric pattern recognition analysis has been limited to using conventional time-domain (TD) feature set [four time domain statistics proposed by Hudgins et al. (9) and auto-regressive (AR) + root mean square (RMS) feature set (a combination of AR coefficients and RMS amplitude) (5)]. Assessment of EMG features for neurological injury patients might be promising to improve myoelectric pattern recognition performance, particularly given the neurologic injury induced muscle impairments (such as weakness, spasticity, and abnormal coactivation) (13).

Time–frequency analysis has been developed as a useful tool for processing non-stationary biosignals (such as EMG). Time–frequency representations of surface EMG such as using wavelet packet transform (WPT) can also be applied in myoelectric pattern recognition, as demonstrated in amputees or able-bodied subjects (10, 14, 15). In the current study, the utility of applying WPT to stroke subjects was examined. The WPT is able to generate a redundant set of subspaces arranged in a binary tree structure with any designed depth/resolution, where the input signal can be accordingly decomposed. Performing wavelet packet analysis of surface EMG recordings from paretic muscles has several advantages. For example, its high resolution in both time and frequency domains makes it feasible to produce a sufficient number of features, from which those highly associated with different movement intentions of the affected limb (i.e., discriminable features) can be easily selected *via* a best basis selection approach to maximize the pattern separability (15, 16). Moreover, such a feature selection approach can be expanded for selecting surface EMG channels (myoelectric control sites) from the high-density surface EMG recordings (by adopting the same discriminant measure). The advantages of the WPT analysis for myoelectric pattern recognition and channel selection were demonstrated for stroke patients. These findings provide useful information for developing a pattern recognition-based myoelectric control system for stroke rehabilitation.

## Methods

### Dataset Description

The dataset used in this study was selected from a database previously reported in Zhang and Zhou (4), which was approved by the Institute Review Board of Northwestern University (Chicago, IL, USA). This database included high-density surface EMG recordings from 12 chronic stroke subjects with hemiparesis during their performance of different functional movements involving the affected upper limb, notably the affected hand. The detailed demographic information and clinical assessment for the stroke subjects can be found in Ref. (4). All subjects gave their informed consent before the experiment. Table 1 displays demographics and clinical information of all stroke subjects in detail.

**Table 1:** **Subject demographics and clinical information**.

Subject #	Age	Sex	Duration	Paretic	FMUE	C–M hand
1	59	F	13	L	28	2
2	56	M	23	L	15	2
3	67	M	8	L	20	4
4	63	F	7	R	19	2
5	45	M	6	L	58	5
6	58	F	2	R	23	2
7	64	M	8	L	38	2
8	61	M	7	R	56	4
9	65	M	15	L	20	2
10	46	M	13	L	52	3
11	81	M	17	L	28	2
12	71	F	22	R	22	3

*Duration, years post stroke; paretic, the side of hemiparesis; FMUE, the Fugl-Meyer assessment scale of the paretic upper-extremity (total score: 66); C–M hand, the hand impairment part of the Chedoke–McMaster stroke assessment scale (from 1 to 7)*.

During the experiment, each subject was instructed to perform 20 functional movements using the affected upper limb, namely, wrist flexion/extension, wrist supination/pronation, elbow flexion/extension, hand open/close, thumb flexion/extension, index finger flexion/extension, finger 3–5 flexion/extension, fine pinch, lateral pinch, tip pinch, gun posture, and ulnar wrist down/up. A video demonstration of each movement was used as a guide for subjects to follow and perform the movement. The experiment protocol comprised of 20 trials, each trial consisting of 5 repetitions of the same movement. For each repetition, the subject was asked to hold the muscle contraction for roughly 3 s and then relaxed for a rest period of 5–20 s.

**Figure 1:**
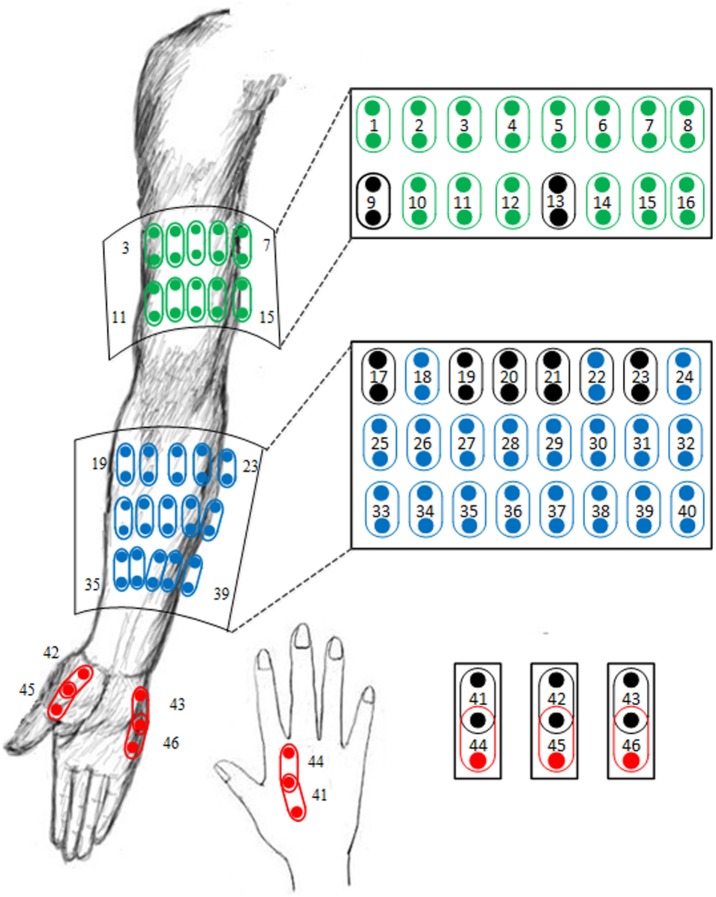
**Illustration of the electrode placement for 46-channel bipolar sEMG signal recordings derived from 89-channel monopolar sEMG database**. The 10 electrode channels marked in a black/darker color are included in an empirically defined channel set.

The high-density surface EMG signals in the original database were recorded *via* 89 monopolar surface electrodes placed on the affected upper arm, forearm, and hand muscles. A Refa EMG recording system (TMS International BV, Netherlands) with a band-pass filter between 20 and 500 Hz was used for multi-channel EMG recording at a sampling rate of 2 kHz per channel. Due to improved myoelectric classification performance and more clinical relevance compared with monopolar configuration, 46-channel bipolar surface EMG data were produced from the original 89-channel EMG recordings. The detailed information about the electrode formation and single spatial differential filter is shown in Figure 1. Besides, 10 bipolar channels, namely, the channel 9, 13, 17, 19–21, 23, and 41–43 were selected from the 46 channels to form a channel set. The selection of such a channel set was in accordance with electrode sites frequently used in many previously reported myoelectric control systems (8). These channels were regarded to target at primary muscles with high relevance to functional movements of the upper limb, as marked in a black/darker color in Figure 1. In this study, such an empirically defined channel set was compared with all 46 high-density channels or a number of optimally selected channels in terms of myoelectric control performance.

For the recorded signals, the onset and offset of a voluntary EMG activity segment corresponding to each repetition of muscle contraction were determined first as described in Ref. (4). For each repetition of muscle contraction, the EMG activity segment in a form of multiple channels was further segmented into a series of overlapping analysis windows with a window length of 256 ms and an overlapping rate of 75% for two consecutive windows. Consequently, the following feature extraction and classification procedures were performed on these analysis windows.

### Feature Extraction Using WPT

The WPT was a generalized version of classical wavelet decomposition method that offers a multi-resolution and time–frequency analysis of non-stationary, such as biomedical signals (17, 18). Define the original signal space as Ω_0,0_. The WPT is able to split the signal into an approximation (in subspace Ω_1,0_) and a detail (in subspace Ω_1,1_). Each approximation or detail obtained from the top-level, supposed in the subspace Ω*_j_*_,_*_k_*, can be further split into a new approximation and a new detail, located in two orthogonal subspaces Ω*_j_*_+1,2_*_k_* and Ω*_j_*_+1,2_*_k_*_+1_, respectively. This process can be iteratively performed to a targeted depth *J*. Here, *j* is a scale index ranging from 0 to *J*, and *k* represents subband index within the scale, ranging from 0 to 2*^j^* − 1. Consequently, the WPT generates a binary tree structure of subspaces spanned by a set of bases, to which a signal can be mapped for multi-resolution analysis. Such a characteristic allows WPT to be successfully applied to feature extraction in the fields of pattern recognition (14, 15, 18).

In this study, the WPT with the five-order symmlet wavelet was first applied to each channel of an analysis window for feature extraction. The five-order symmlet wavelet was selected from many mother wavelet functions frequently used in previous reports (10, 14, 15) and was further determined by some pretests in terms of classification performance. The WPT depth is also an important factor for WPT analysis. It is acknowledged that a small depth cannot yield sufficient resolution for extracting effective features, whereas a large depth leads to much more computational complexity. By considering this trade-off, the WPT depth of 3 or 4 has been recommended by previous studies (15, 19). The WPT depth of 4 was chosen in this study after some pretests, thus producing 30 subspaces in total. After the WPT, the energy values of all 30 subspaces were calculated as potential features (refer to feature selection approach in the following section), where the energy of each subspace was defined as a logarithmic value of the summation of the squares of all wavelet packet coefficients in the subspace. The logarithmic transform was chosen for showing better performance of classification after some tests.

### Feature Selection Using Best Basis Selection

The WPT binary tree yielded a redundant set of subspaces due to the subspace overlap in frequency axis. Afterward, the features extracted from all subspaces were regarded to carry redundant information. A great number of redundant features were likely to impose high computational cost and compromise classification performance. For application of the WPT analysis to feature extraction or pattern recognition, a best basis is usually chosen to maximize the class separability in terms of a proper discriminant measure. To achieve this goal, a feature selection procedure relying on a best basis selection algorithm is necessary. In this study, the algorithm was designed to choose the best set of subspaces from the WPT binary tree, since each subspace produced a potential feature. To determine the best subspace, Fisher’s class separability index (FCSI) described in Ref. (20) was employed as the discriminant measure, which is introduced below.

Suppose that ${\left\{x_{i,\left(j,k\right)}^{\text{(}c\text{)}}\right\}}_{i=1}^{N_{c}}$ represents a set of energy features extracted from the subspace Ω*_j_*_,_*_k_* of the training signals belonging to class *c* (1 ≤ *c* ≤ *C*, here *C* = 20), where *N_c_* is the number of samples (i.e., analysis windows) in class *c*.

For each subspace, the mean and variance of these features grouped by class can be calculated as

(1) $${\bar{m}}_{j,k}^{\left(c\right)}=\frac{1}{N_{c}}\sum_{i=1}^{N_{c}}{\left\{x_{i,\left(j,k\right)}^{\text{(}c\text{)}}\right\}}_{i=1}^{N_{c}},$$

(2) $$\underset{1\leq i\leq N_{c}}{\mathrm{var}}\;\left(x_{i,\left(j,k\right)}^{\left(\text{c}\right)}\right)=\frac{1}{N_{c}}\sum_{i=1}^{N_{c}}{\left(x_{i,\left(j,k\right)}^{\left(c\right)}-{\bar{m}}_{j,k}^{\left(\text{c}\right)}\right)}^{2}.$$

Here, the operator var*_i_*(·) is defined to calculate the variance of a set of constant variables indexed by *i*. Thus, the FCSI, for the subspace Ω*_j_*_,_*_k_*, is finally defined as

(3) $$\text{FCSI}=\sum_{p=1}^{K-1}\sum_{q=p+1}^{K}\frac{{\left|{\bar{m}}_{j,k}^{\left(p\right)}-{\bar{m}}_{j,k}^{\left(q\right)}\right|}^{2}}{\underset{1\leq i\leq N_{p}}{\mathrm{var}}\;\left(x_{i,\left(j,k\right)}^{\left(p\right)}\right)+\underset{1\leq i\leq N_{q}}{\mathrm{var}}\;\left(x_{i,\left(j,k\right)}^{\left(q\right)}\right)}.$$

where *p* and *q* represent the indices of two different classes. Generally, a higher value of FCSI indicates higher degree of class separability. The best basis selection algorithm using FCSI is able to rank the features and make it practical to choose a subset of these regarded as being most discriminant.

In this study, feature selection approach was independently performed on each channel. Many previous studies regarding wavelet packet features also took the same procedure (10, 21, 22). For each channel, the number of selected subspaces/features needed to be carefully determined. It should be acknowledged that inadequate number of features may not guarantee the classification performance, whereas too many features lead to much computational cost. Considering such a trade-off, we set the number of subspaces/features per channel to 12 after performing sensitivity analysis (in terms of classification accuracy) by varying the feature number per channel from 1 to 25. Finally, the features from all channels were further concatenated as a high-dimensional feature vector for each analysis window.

### Feature Dimensionality Reduction and Classification

Even with the above feature selection procedure, the high-density surface EMG recordings still resulted in very high-dimensional feature vectors (i.e., 552-dimensional feature vectors with 12 best bases for each of 46 channels). In this case, feature dimensionality reduction is required to ensure the generalization capability of a classifier (23). In this study, uncorrelated linear discriminant analysis (ULDA) was used to reduce the feature dimension, which minimizes within-class distance and maximize between-class distance by an optimal transformation (24).

After the feature dimensionality reduction, linear discriminant classifier (LDC) was employed in this study. The LDC is able to model the within-class density of each class as a multi-variant Gaussian distribution and gives decisions of unknown samples by using the maximum a posteriori probability (MAP) rule and Bayesian principles (9, 25). The LDC was used due to its ease of implementation and efficient classification performance (4, 8).

In this study, the pattern classification was conducted in a user-specific manner, where both training dataset and testing dataset were derived from the same stroke subject. A fivefold cross-validation was conducted to evaluate the classification performance. This indicated that the EMG data from any four repetitions of muscle contraction were selected and assigned as training dataset, while the EMG data from the remaining repetition were used to form the testing dataset. The classification performance for each subject was evaluated as classification accuracy, which was calculated as the percentage of correctly classified windows over all the testing windows including all movement patterns over testing dataset. These window numbers were summed up over all fivefold tests for each subject.

For the performance comparison, the routine TD feature set including four statistics of the surface EMG signals, namely, mean absolute value (MAV), the number of zero crossing (ZC), the slope sign change (SSC), and the waveform length (WL), was also employed during the tests. The TD feature set was used in a similar way as previous studies that all TD features from all the considered channels were concatenated to form a feature vector for each analysis window. The same feature dimension reduction approach using ULDA was applied as well before LDC classifier implementation.

### Channel Selection

The use of FCSI for quantifying the discriminating power of features was further extended to channel selection from high-density surface EMG recordings. After the feature extraction and selection methods introduced above, a subset of features was determined for each channel and used to form a vector representing the most discriminable information from that channel. In order to perform channel selection, it was necessary to assess the discriminating power of feature vectors rather than scalars. Thus, the FCSI was accordingly modified as follows.

Here, let ${\left\{{\text{x}}_{i,l}^{\left(c\right)}\right\}}_{i=1}^{N_{c}}$ be a set of feature vectors extracted from the *l*-th channel of the training data belonging to class *c*. The mean of these feature vectors, originally defined in Eq. 1, needs to be modified, and their variance, namely $\underset{1\leq i\leq N_{c}}{\mathrm{var}}\;\left({\text{x}}_{i,l}^{\left(\text{c}\right)}\right)$, is further defined to be the summation of all variances calculated along any single dimension of the vector, as depicted in Eqs 4 and 5.

(4) $${\bar{\text{m}}}_{l}^{\left(\text{c}\right)}=\frac{1}{N_{c}}\sum_{i=1}^{N_{c}}{\text{x}}_{i,l}^{\left(c\right)},$$

(5) $$\underset{1\leq i\leq N_{c}}{\mathrm{var}}\;\left({\text{x}}_{i,l}^{\left(\text{c}\right)}\right)=\underset{1\leq i\leq N_{c}}{\mathrm{var}}\;\left(x1_{i,l}^{\left(\text{c}\right)}\right)+\underset{1\leq i\leq N_{c}}{\mathrm{var}}\;\left(x2_{i,l}^{\left(\text{c}\right)}\right)+\cdots +\underset{1\leq i\leq N_{c}}{\mathrm{var}}\;\left(xd_{i,l}^{\left(\text{c}\right)}\right),$$

where **x** = [*x*1,*x*2, …,*xd*]*^T^* denotes a *d*-dimensional vector. Thus, the FCSI, for the *l*-th channel, can be finally computed *via*

(6) $$\text{FCSI}=\sum_{p=1}^{K-1}\sum_{q=p+1}^{K}\frac{{\left|{\bar{\text{m}}}_{l}^{\left(p\right)}-{\bar{\text{m}}}_{l}^{\left(q\right)}\right|}^{2}}{\underset{1\leq i\leq N_{p}}{\mathrm{var}}\;\left({\text{x}}_{i,l}^{\left(p\right)}\right)+\underset{1\leq i\leq N_{q}}{\mathrm{var}}\;\left({\text{x}}_{i,l}^{\left(q\right)}\right)},$$

where *p* and *q* represent the indices of two different classes again. Similarly, a higher FCSI value indicates a higher degree of class separability for a certain channel. Following the strategy of feature selection using FCSI, a subset of optimal channels can be selected by ranking the channels using FCSI. This channel selection approach was termed as FCSI method in this study.

Channel selection has also been conducted in previous studies (25, 26) to assess the myoelectric pattern recognition performance using a reduced number of EMG channels selected from high-density signal recordings. A straightforward algorithm, termed as sequential feedforward selection (SFS), was often used, which iteratively adds the most informative channels in terms of classification accuracy. In the first iteration of this algorithm, each of all candidate channels is independently used and the channel producing the highest classification accuracy was selected to be the first optimal channel. During the next iteration, the previously selected channels were combined with each of the other channels to form a new subset sequentially, and the subset producing the highest classification accuracy was determined. This procedure can be iteratively performed when meeting a desired number of selected EMG channels. Note that the SFS directly uses the classification accuracy as the criterion, which conventionally requires classifier training and testing procedures in each iteration. Thus, the channels selected by the SFS algorithm are more likely to be overfitted to the testing data with limited generalization ability. In order to avoid such overestimated performance in some degree, the SFS algorithm used in this study was performed only on the training dataset. This required the original training dataset consisting of four repetitions of muscle contraction to be further divided into two parts, one consisting of three repetitions for SFS training and the other consisting of the remaining repetition for SFS testing. To evaluate the classification performance with the channels selected by the SFS algorithm, a classifier was implemented with all the four repetitions (used for SFS) as training dataset and the remaining fifth repetition (which was not used for SFS) as testing dataset.

The channel selection using FCSI is able to independently choose a subset of best channels in any size *m*. It should be acknowledged that the *m* best channels may not be the best *m* channels. By contrast, the standard SFS algorithm offers a practical way of selecting a subset of appropriate channels by taking the effect of channel combination into account, but it conventionally suffers from the overfitting problem. By taking advantage of both methods to overcome its own drawbacks, a novel channel selection method named FCSI + SFS was proposed in this study. For clarity, the FCSI + SFS algorithm can be briefly described as follows:

(a) Initialize a candidate channel set Φ = {*l*|*l* = 1, 2, …, *L*} and a selected channel set ψ = empty, where *L* denotes the total channel number.
(b) For any channel *l* in Φ, calculate its FCSI value *via* Eqs 4–6.
(c) Choose the channel *l_m_* that yields the highest FCSI value among channels in Φ and then move the channel *l_m_* from Φ to ψ.
(d) For any remaining channel *l* in Φ, combine the channel *l* with all channels in ψ and calculate the FSCI value of their combination *via* Eqs 4–6. Note that in this case, the feature vector **x** is formed by concatenating features from all combined channels. If applicable, the high dimensionality of these feature vectors was reduced by ULDA prior to the FSCI evaluation.
(e) Choose the channel *l_m_* that yields the highest FCSI value, when it is combined with all channels in ψ, and then move the channel *l_m_* from Φ to ψ.
(f) Repeat the steps (d) and (e), until the size of the selected channel set ψ reaches into a preset number.

Consequently, the performance of the proposed FCSI and FCSI + SFS algorithms was examined and compared with solely using the SFS algorithm for channel selection. To ensure a fair comparison, all the three algorithms selected their respective desired channels using the training dataset (i.e., four repetitions), while the classification performance of the selected channels was evaluated using the fifth one (not involved in channel selection process) as testing dataset for the classifier.

## Results

### Feature Selection and Classification

**Figure 2:**
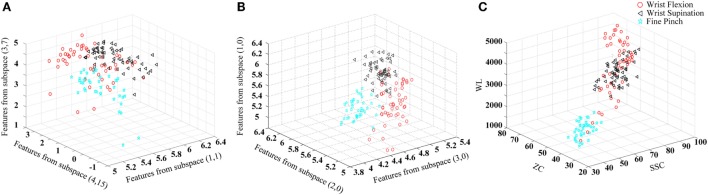
**Illustration of the effect of FCSI values on feature separability**. Three upper limb movements (wrist flexion, wrist supination, and fine pinch) in the 18-th channel from Subject 3 are used as an example to produce the scatter plots. The three-dimensional coordinate axes stand for feature values. **(A)** Three features with the lowest FCSI values; **(B)** three features with the highest FCSI values; and **(C)** three TD features (WL, ZC, and SSC) used for comparison.

An example of the effectiveness of FCSI for quantifying the discriminating power of features is shown in Figure 2, where the distribution of features for three representative classes were demonstrated in three scatter plots: (a) with the lowest FCSI values, (b) with the highest FCSI values, and (c) from three TD parameters (WL, ZC, and SSC). From the visual inspection, it can be found that the features determined by three highest FCSI values reflect good class separability in the figure, whereas such separability was not observed for features with lowest FCSI values or three TD features.

Following the wavelet packet feature extraction and selection using FCSI, along with LDC classification, pattern recognition analysis was implemented in a user-specific manner for all 12 stroke subjects. Table 2 summarizes the classification performance in terms of overall accuracy for identifying 20 intended upper limb movement, when both the WPT-based method and TD feature extraction method were applied to the EMG data consisting of 46 high-density channels or 10 predefined channels, respectively. A two-way repeated-measure ANOVA was applied on the classification accuracies, with the channel number (high-density 46 and 10) and feature set (WPT and TD) both considered as within-subject factors, in order to examine their effect. It can be unsurprisingly observed that high classification accuracies above 95% were achieved for almost all subjects when the 46 high-density channels were totally used, regardless of the feature extraction methods. By contrast, the use of predefined 10 channels led to a performance compromise with an averaged accuracy of 91.15% for the TD features and 92.91% for the WPT features, respectively. An overall significant effect of both channel number (*F* = 14.597, *p* = 0.003) and feature set (*F* = 10.031, *p* = 0.009) on classification accuracy was revealed by the ANOVA. In this case, the WPT-based feature extraction approach showed superior performance to the routine TD feature extraction method by about 2% accuracy improvement with statistical significance.

**Table 2:** **Classification accuracy (unit: %) in stroke subjects when both TD and WPT features were extracted from the EMG data of 46 high-density channels and 10 predefined channels, respectively**.

Subject #	46 high-density channels		10 predefined channels	
	TD	WPT	TD	WPT
1	94.36	98.74	82.89	86.57
2	91.15	95.75	80.61	82.46
3	94.07	98.56	93.47	89.34
4	87.36	98.00	82.93	87.69
5	96.81	94.22	96.73	98.49
6	95.02	98.61	86.56	86.65
7	99.65	100.0	94.67	96.56
8	99.58	100.0	96.39	99.47
9	93.63	98.96	95.94	94.90
10	97.84	99.78	86.80	96.26
11	99.32	99.78	98.60	97.95
12	100.0	100.0	98.20	98.60
Average	95.73 ± 3.90	98.53 ± 1.82	91.15 ± 6.68	92.92 ± 5.95

*TD, the time domain feature set; WPT, the proposed feature set using wavelet packet transform*.

### Channel Selection

**Figure 3:**
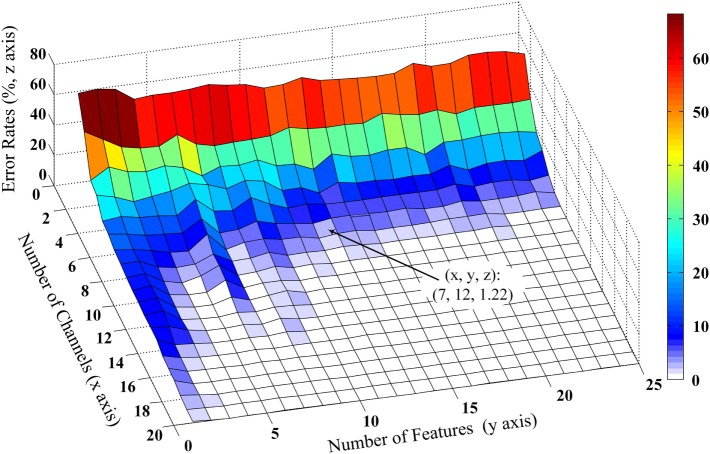
**Effect of number of optimal wavelet basis on the channel selection performance from Subject 2**. The three-dimensional *x*, *y*, and *z* coordinate axes stand for number of channels, number of features, and error classification rates evaluated by LDC classifier. The number of optimal wavelet basis can be determined based on features first reaching the minimum error rate and the trade-off of computational cost and classification performance.

The performance of the proposed method for selecting an appropriate subset of channels was further examined. Admittedly, the performance of myoelectric pattern recognition is sensitive to both the channel number and the number of extracted features per channel. By changing both factors, their effect on the classification performance was simultaneously examined using the FCSI algorithm. Figure 3 shows a representative example from Subject 2 illustrating how the classification performance (as described by error rate) changes in the extracted/selected feature number per channel varying from 1 to 25 and the channel number varying from 1 to 20. It can be observed that very low (approximately 1 or 2) feature number per channel or channel number could not produce high classification performance (low error rate) and the increase of channel number played a critical role in performance improvement. Similar findings were observed for the other subjects. Considering the trade-off between classification performance and practicability (i.e., low computational cost and reduced number of channels), the feature number was set to be 12, producing an error rate of 1.22% for Subject 2 when the channel number was reduced to 7. This confirmed our setting of feature number per channel to 12 in both previous and following data analyses.

**Figure 4:**
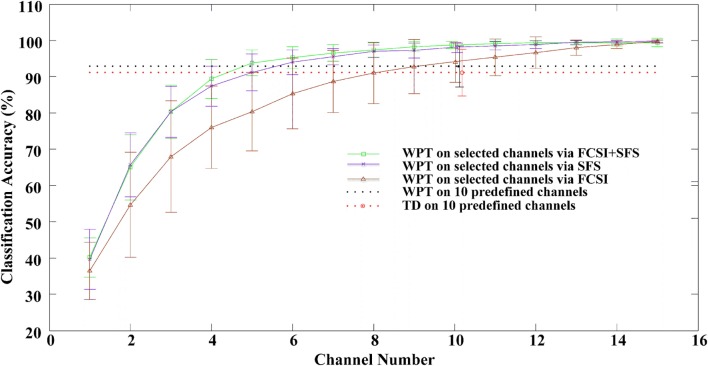
**The classification performance as a function of number of channels selected *via* the FCSI, SFS, and FCSI + SFS methods, respectively**. For each subject, the classification accuracies were derived from the testing dataset. The classification accuracies from all 12 subjects were averaged and plotted with SD error bars. The classification accuracies derived from applying both WPT and TD features to 10 predefined channels are indicated as two horizontal dashed lines.

After the feature number per channel was appropriately determined, the performance of three channel selection algorithms was evaluated. Figure 4 reports the classification accuracies averaged across 12 stroke subjects, when the EMG channels were progressively selected using FCSI, SFS, and FCSI + SFS, respectively. When applying WPT and TD feature extraction methods on 10 predefined channels, in addition, the achieved classification accuracies are indicated as two horizontal dashed lines in Figure 4 for comparison purpose. It can be found that each of the three algorithms yielded a similar increasing trend of classification accuracy when the channel number increased. The classification accuracy increased rapidly to approximately or over 90% at channel number ranging from 1 to 10 and then remained almost steady or slightly increased with further channel number increasing. The proposed FCSI + SFS method demonstrated its superior performance to the other two with highest average accuracies. Specifically, by using 10 optimally selected channels as compared with the 10 predefined channels, improved classification performance was obtained. Meanwhile, the use of only 5 channels optimally selected by either FCSI + SFS or SFS algorithm was found to produce classification performance comparable to that of using 10 predefined channels. Furthermore, a series of bivariate Pearson’s correlation analyses were conducted to further examine the effect of subjects’ clinical information (including years post stroke, FMUE, and C–M hand scores) on the classification accuracies derived from the use of any channel number (high-density 46, predefined 10, or optimally selected 10 channels by FCSI_SFS or SFS) along with any feature set (WPT or TD), respectively. No significant correlation was found between any clinical information and the classification accuracy (*p* > 0.058) except the correlation between the FMUE score and the classification performance with WPT feature extracted from 10 predefined channels (correlation coefficient *R* = 0.651, *p* = 0.022).

Table 3 shows the first 10 selected channels for each subject using the 3 methods. It was found, as would be expected, that the selected channels were different across subjects even using the same method. For each subject, the selected channels also varied when three methods were performed, respectively. However, for each subject, several channels (marked in bold numbers, though, with varying order of selection) were commonly selected using any of the three algorithms.

**Table 3:** **List of the first 10 selected channels for all 12 stroke subjects using 3 channel selection methods, respectively**.

Subject #	FCSI + SFS	SFS	FCSI
	Channel combination	Channel combination	Channel combination
1	(**46**, 6, **7**, 29, 40, 5, 26, 27, 20, 44)	(6, **46**, 2, 27, 3, 17, 36, 29, 26, **7**)	(19, 11, 3, **46**, 43, 20, 2, 12, **7**, 21)
2	(**10**, **17**, **44**, 1, 6, 13, 34, 36, 24, 8)	(4, 25, **44**, 5, 6, **10**, 31, **17**, 7, 27)	(2, **44**, 13, **17**, 25, **10**, 1, 5, 38, 16)
3	(22, 38, **45**, 12, 18, 43, 44, 31, 2, 25)	(18, 30, **45**, 5, 43, 25, 26, 29, 27, 1)	(**45**, 30, 42, 31, 41, 1, 38, 8, 23, 22)
4	(30, 42, 46, 22, 12, 32, **4**, 25, 35, 21)	(**4**, 45, 5, 35, 39, 22, 19, 6, 26, 20)	(33, 32, 28, 25, 20, 17, 19, 12, **4**, 40)
5	(**37**, 42, 17, 21, 44, 24, 34, **27**, 46, 9)	(24, **37**, 43, **27**, 31, 35, 18, 10, 32, 4)	(42, 45, **37**, 3, 38, 4, 9, 44, 41, **27**)
6	(**46**, 24, 37, 40, 5, 34, 30, 6, 42, 43)	(**46**, 30, 18, 40, 32, 20, 24, 17, 34, 16)	(41, 44, 43, **46**, 27, 5, 28, 13, 38, 18)
7	(37, 43, 46, 31, 41, 17, 22, 24, 13, 19)	(37, 23, 17, 34, 27, 30, 35, 39, 15, 26)	(1, 13, 16, 9, 10, 5, 8, 14, 6, 27)
8	(4, 43, 41, 38, 17, **11**, 5, 32, 23, 3)	(17, 19, 23, 29, 26, 13, 39, 8, **11**, 6)	(5, 4, 13, 22, 12, 3, 2, **11**, 8, 25)
9	(21, 38, **22**, **44**, 24, 37, 9, 29, 42, 17)	(37, **22**, 20, 41, 10, **44**, 35, 21, 12, 23)	(**44**, **22**, 17, 4, 2, 26, 10, 40, 32, 7)
10	(10, 31, **33**, 45, 16, 30, 26, 44, 11, 38)	(30, 18, 15, 28, 25, 23, **33**, 34, 26, 38)	(10, 16, 8, 9, 1, **33**, 7, 15, 32, 2)
11	(**31**, **17**, 40, 30, 28, 45, 42, 43, 24, 23)	(31, 25, 13, 36, 4, 16, 18, 5, 21, **17**)	(42, 45, 46, 43, 25, 37, 23, 39, **31**, **17**)
12	(**24**, 21, 5, 16, 43, 44, 12, 17, 32, 38)	(**24**, 5, 37, 27, 9, 1, 35, 3, 7, 6)	(32, 43, 44, 25, 41, 37, **24**, 4, 2, 12)

*The bold numbers represent commonly selected channels using any of the three methods for each subject*.

## Discussion

Myoelectric pattern recognition has great potential for implementing interactive control of assistive robotic devices, which is of particular importance for restoration of dexterous arm/hand functions. Previous high-density surface EMG pattern recognition analysis using conventional TD or AR + RMS features has revealed that substantial neural control information can be readily extracted from paretic muscles of stroke patients. In the current study, a feature extraction method based on WPT was re-examined and applied to high-density surface EMG signals from stroke subjects for improved myoelectric pattern-recognition performance. Taking advantage of the classic wavelet packet feature extraction and selection approach, a novel channel selection method was furthermore developed to determine a practical number of appropriate EMG channels for maintaining high classification accuracies, an issue particularly important for implementing a practical myoelectric control system.

The FCSI was used in this study to quantify the discriminating power of each feature or wavelet packet basis/subspace where the feature was derived. There have been different algorithms or criteria for determining the best basis or subspace in WPT analysis (15, 20). For pattern recognition analysis, the adopted criterion is preferably associated with class separability. The FCSI is such a criterion that is able to measure the class separability of a feature or feature vector, more specifically, in almost the same way as the LDC classifier does. The FCSI was used to determine the most discriminating features from those produced by WPT analysis in various time–frequency scales. Due to the advantages of time–frequency resolution provided by the WPT as well as the FCSI analysis, the wavelet packet feature extraction and selection approaches demonstrated improved performance, as compared with the previously used conventional time domain or frequency domain feature sets, especially for subjects with relatively high levels of impairments. For example, 5 of 12 stroke subjects (i.e., Subjects 1, 2, 3, 4, and 9) produced relatively low classification accuracies below 95.0%, respectively, when the TD feature set was applied, whereas improved accuracies above 95% (Table 2) were achieved for these subjects using the wavelet packet features. It is worth noting that the TD and AR features were often employed for myoelectric pattern recognition for amputee subjects toward prosthesis control, which can achieve comparable performance to more complicated features including wavelet packet features (8, 9, 25). In contrast, the advantages of wavelet packet feature extraction and selection appeared more evident in processing stroke data, presumably due to the fact that the residual muscles of an amputee subject are neurologically intact, whereas the paretic muscles of stroke subjects usually suffer from different symptoms such as weakness, spasticity, etc. Due to the fact that neural control information delivery is hampered by injuries to neuromuscular pathways after stroke, more complicated features (e.g., WPT features) are likely to emerge their advantage in characterizing such paretic EMG signals, which was demonstrated in this study.

The FCSI used for WPT feature selection was further extended for channel selection, in combination with the SFS method. The combined FCSI + SFS method demonstrated superior performance for channel selection in terms of classification performance than solely using the SFS or the FCSI method. Using the FCSI rather than the direct classification accuracy in combination with the SFS algorithm avoids repeated training and testing of a classifier (required for each iteration). Furthermore, in this study, the channel selection procedure and the performance testing procedure were not based on the same datasets in order to overcome the overfitting problem. Besides, when the same number (e.g., 10) of channels were employed, the channels selected *via* an optimal algorithm (e.g., FCSI + SFS) yielded higher classification accuracies across all 12 stroke subjects than those predefined electrode sites. In addition, with the WPT feature set, the correlation analyses revealed dependence of the classification performance on the FMUE score with statistical significance (*p* = 0.021) when a set of predefined 10 channels were used. Such dependence disappeared when high-density 46 channels or optimally selected 10 channels were adopted (*p* > 0.021), indicating that those subjects with relatively high levels of impairments (i.e., low clinical assessment scores) had substantial improvement of classification performance. The channel selection analysis not only confirms previous findings in Ref. (4, 27) that it is feasible to use a small number of EMG channels (rather than a high-density electrode array) for decoding sufficient neural control information from paretic muscles but also indicates the necessity of determining appropriate control site locations (rather than predefined channels) for improved classification performance. Therefore, effective algorithms, such as the FCSI + SFS, reported in this study are of critical demand for developing a practical myoelectric control system, particularly for stroke users.

When examining the selected channel index, it was found that the selected channels were different among 12 stroke subjects even using the same channel selection method, primarily due to individual subject difference following stroke (such as impairment nature and level, recovery status, daily activity, etc.). It confirms our previous suggestion (4) that the myoelectric pattern recognition should be designed or conducted in a user-specific manner. The designed system may include appropriate EMG features and channels (e.g., electrode number, location, configuration, etc.) that maximize the classification accuracy to enhance its usability for stroke subjects with any impairment level, while its suitability for real time application (such as computational cost, adaptability to slight electrode movement, etc.) should also be considered. We acknowledge that the examined WPT-based feature extraction and selection approach may induce relatively higher computational complexity than using conventional TD feature set. Even so, the WPT method is still very practical for real-time implementation demonstrated by an enormous number of previous studies (10, 14, 28). Also, the choice of the target movements or controlled function should consider subject need and classification performance. Although high-density surface EMG recording contains much redundant information for myoelectric pattern recognition analysis, it provides a very useful and essential way to optimize the myoelectic control system designed for individual stroke patients. In this regard, the high-density sEMG recording, along with effective channel selection, can be designed as a necessary calibration procedure. Such a procedure is recommended to be conducted just once, rather than regularly, during the prescription of the myoelectrically controlled robotic training for stroke patients with different impairment levels.

## Conclusion

In this study, a feature extraction method based on WPT was applied to myoelectric pattern recognition analysis in stroke survivors. By processing high-density surface EMG recordings from paretic muscles of 12 stroke subjects, the WPT features achieved an improved performance for classification of 20 different arm/hand movements compared with the conventional TD EMG features. Furthermore, a novel channel selection method was developed by combining the FCSI and the SFS analyses, which can effectively determine a small number of appropriate EMG channels without significantly compromising the classification performance achieved from high-density surface EMG. These novel feature extraction and channel selection analyses confirm substantial neural control information available in paretic muscles of stroke survivors, and moreover, demonstrate the feasibility of extracting such information with a practical number of EMG channels. The findings are helpful for development of myoelectric control systems for stroke rehabilitation.

## Author Contributions

DW analyzed the data, interpreted the results, and wrote the first draft of the manuscript. XZ designed the study and performed all stages of the study including data collection, analysis, interpretation, and substantial revision of the manuscript. XG, XC, and PZ participated in data analysis and interpretation and revised the manuscript. All the authors approved the final version of the manuscript.

## Conflict of Interest Statement

The authors declare that the research was conducted in the absence of any commercial or financial relationships that could be construed as a potential conflict of interest.
